# Multiple Toxin-Antitoxin Systems in *Mycobacterium tuberculosis*

**DOI:** 10.3390/toxins6031002

**Published:** 2014-03-06

**Authors:** Ambre Sala, Patricia Bordes, Pierre Genevaux

**Affiliations:** Laboratoire de Microbiologie et Génétique Moléculaire (LMGM), Centre National de la Recherche Scientifique (CNRS), Université Paul Sabatier, 118 route de Narbonne, Toulouse 31062, France

**Keywords:** toxin-antitoxins, molecular chaperones, proteases, persistence

## Abstract

The hallmark of *Mycobacterium tuberculosis* is its ability to persist for a long-term in host granulomas, in a non-replicating and drug-tolerant state, and later awaken to cause disease. To date, the cellular factors and the molecular mechanisms that mediate entry into the persistence phase are poorly understood. Remarkably, *M. tuberculosis* possesses a very high number of toxin-antitoxin (TA) systems in its chromosome, 79 in total, regrouping both well-known (68) and novel (11) families, with some of them being strongly induced in drug-tolerant persisters. In agreement with the capacity of stress-responsive TA systems to generate persisters in other bacteria, it has been proposed that activation of TA systems in *M. tuberculosis* could contribute to its pathogenesis. Herein, we review the current knowledge on the multiple TA families present in this bacterium, their mechanism, and their potential role in physiology and virulence.

## 1. General Overview of TA Systems in *M. tuberculosis*

Toxin-antitoxin (TA) systems are small genetic modules originally discovered as plasmid-borne loci that promote plasmid maintenance in bacterial populations by killing daughter cells devoid of the TA encoding plasmid [1]. TA loci were subsequently identified in many bacterial and archeal chromosomes, thus suggesting alternative functions [2]. TA systems are typically composed of a protein toxin and a more labile antagonistic antitoxin, which can be a protein or non-coding RNA [1]. Under certain circumstances, including environmental stress, plasmid loss, or bacteriophage infection, the less stable antitoxin is rapidly degraded and the free active toxin is now capable of targeting essential cellular processes such as DNA replication, cell wall synthesis, cell division or translation, thus leading to growth inhibition and eventually cell death. Growth inhibition by toxins is often reversible when new antitoxins are available and, accordingly, it has been proposed that activation of TA systems could facilitate bacterial survival until conditions become more favorable [3]. To date, five main types of TA families, with respect to the mode of inhibition of the poisonous activity of the protein toxin, have been described: (*i*) in type I, a small antisense RNA antitoxin specifically inhibits toxin synthesis by interacting with the toxin mRNA; (*ii*) in type II, the antitoxin is a protein that directly binds and inhibits the toxin; (*iii*) in type III the antitoxin is an RNA that directly interacts with the protein toxin; (*iv*) in type IV the antitoxin and the toxin are proteins that have the same target but do not directly interact with each other; and (*v*) in type V the antitoxin is an endoribonuclease, which specifically degrades the toxin mRNA [1,4,5].

Although the cellular roles of chromosomally-encoded TA systems are not well established, they have been involved in several processes, including stabilization of genomic regions, anti-addiction against similar plasmid-borne toxins, defense against phage infection, biofilm formation, control of the stress response, and, especially, bacterial persistence [1,6]. Persister cells are stochastic phenotypic variants of regular cells, which appear randomly and at low frequency in a population and are tolerant to antibiotic treatments due to slow or arrested growth [7]. Remarkably, it was recently shown that stochastic induction of several chromosomal type II TA systems significantly contributes to such persistence phenotype in *E. coli*, thus, indicating a major involvement of TAs in this process [8].

**Figure 1:**
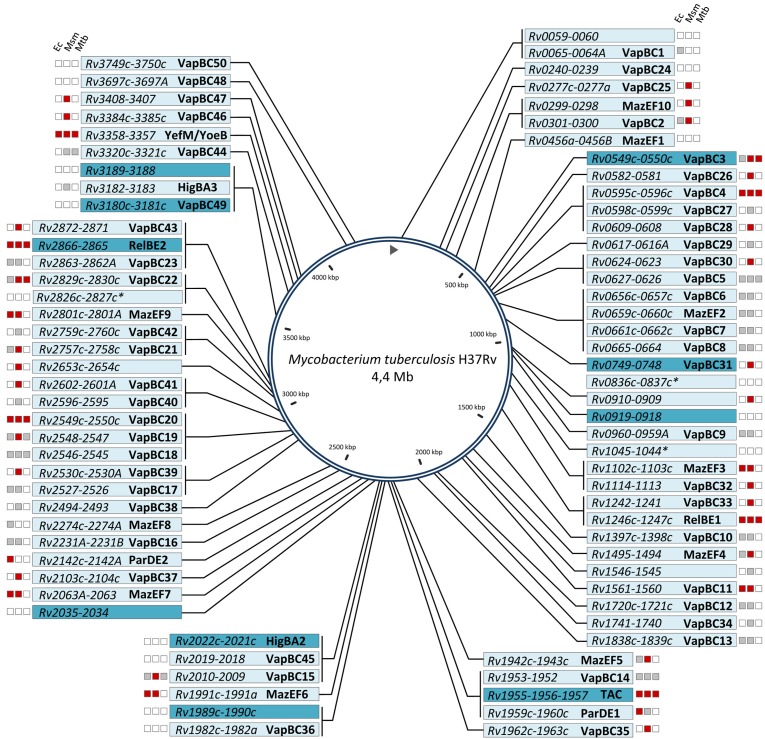
Chromosomal map of *M. tuberculosis* H37Rv TA systems. TA systems are annotated according to the tuberculist database except for VapBC45 (Rv2018-Rv2019), VapBC49 (Rv3181c-Rv3180c), VapBC50 (Rv3750c-Rv3749c), HigBA2 (Rv2022c-Rv2021c), HigBA3 (Rv3182-Rv3183), YefM/YoeB (Rv3357-Rv3358), and MazEF10 (Rv0298-Rv0299). Most of the TA systems depicted here likely belong to type II, expect for those marked with an asterisk, which are putative type IV systems. For each system, the functionality in *E. coli* (Ec), *M. smegmatis* (Msm), and *M. tuberculosis* (Mtb), is depicted: red color stands for “inhibition of growth”, grey for “no inhibition of growth”, and white for “not tested”. The 10 most induced TA systems in drug-tolerant persister cells are highlighted on dark blue background.

Tuberculosis is the deadliest disease due to a single bacterial pathogen, namely *Mycobacterium tuberculosis*. Besides the synergy with the human immunodeficiency virus (HIV) and the emergence of multi and extensively drug resistant strains, one of the limitations to tuberculosis eradication is its ability to persist in human lungs in a dormant state, which is tolerant to the host immune system and to antibiotic treatments. This phenomenon is thought to be responsible for latent tuberculosis, which affects approximately one third of the human population according to the World Health Organization (WHO), and can reactivate later in case of immune depression. The existence of a persistent subpopulation in the active form of the disease is also believed to be responsible, at least in part, for the exceptionally long duration of the anti-tubercular treatment [9]. *M. tuberculosis* possesses a remarkably high number of TA systems in its chromosome when compared to other mycobacteria (Figure 1 and Table 1), and it has been proposed that persistence induced by active toxins could contribute to its pathogenesis [10,11]. Interestingly, a transcriptomic analysis of antibiotic-induced persisters of *M. tuberculosis* revealed that in addition to a general shutdown of metabolic pathways, at least 10 TA systems were significantly up-regulated under these conditions [12], thus, further supporting a potential contribution of TAs in *M. tuberculosis* persistence.

We have identified a total of 79 TA systems (confirmed or putative) in *M. tuberculosis* H37Rv: 67 belonging to six well described type II TA families: VapBC (50 systems), MazEF (10 systems), YefM/YoeB (one system), RelBE (two systems), HigBA (two systems), and ParDE (two systems); one tripartite type II TAC (Toxin-Antitoxin-Chaperone) system controlled by a SecB-like chaperone; three potentially type IV systems; and eight uncharacterized putative TA systems. Note that no other type of TA family was identified in *M. tuberculosis* [13,14]. Most of these systems (63) have been experimentally tested, essentially in *E. coli* and *M. smegmatis*, for growth inhibition by the putative toxin and its neutralization by the putative antitoxin. Among these, 37 were shown to be functional in at least one of the conditions tested (Figure 1) [15,16,17,18,19,20]. This review presents the current knowledge on TA systems in *M. tuberculosis*.

**Table 1:** Toxin-antitoxin systems in representative mycobacterial genomes ^a^.

Genomes	VapBC	MazEF	RelBE	ParDE	YefM/YoeB	HigBA	TAC	Other^b^	Total
*M. tuberculosis* H37Rv	50	10	2	2	1	2	1	11	79
*M. smegmatis* MC^2^155	1	1					1^c^	2	4
*M. marinum* M								1	1
*M. avium* 104	1							2	3
*M. avium paratuberculosis* K10	1		1					1	3
*M. abscessus* ATCC 19977						1		7	8
*M. ulcerans* Agy99	1							1	2
*M. gilvum* PYR-GCK	3		1				1	9	14

Notes: ^a^ Data were collected on TADB (Toxin-Antitoxin Database) or searching for TA homologs using BLASTP on the NCBI server; ^b^ Unclassified TA systems: only one couple of conserved domains found in *M. abscessus* and *M. gilvum*, namely COG2856-Xre, was not present in *M. tuberculosis*; DUF1814-COG5340 was found in *M. marinum*, *M. avium* and *M. gilvum*; COG3832-ArsR was found in all the investigated mycobacterial genomes except from *M. marinum*; one GNAT-RHH and one COG5654-Xre were found in *M. gilvum*. See Part “Other TA modules” for details; ^c^ Indicates that in *M. smegmatis* the putative TAC system is incomplete due to the absence of toxin gene.

## 2. The VapBC Family

The VapBC (Virulence associated protein) family of TAs was first discovered as an addiction system of a virulence plasmid of *Salmonella dublin* [21]. They are the most abundant TA loci in *M. tuberculosis* (Figure 1), defined by a toxin VapC with a PIN domain (homologous to PilT N-terminal domain) found on proteins from all life kingdoms and generally ribonucleases [22]. Most of the VapC toxins tested so far, including VapC1, VapC2, VapC5, VapC11, VapC20, and VapC29 from *M. tuberculosis,* exhibit a ribonuclease activity *in vitro* [16,20,23,24]. However, toxicity of the *M. tuberculosis* VapC4 seems to be induced by stable RNA binding and not by degradation [25]. Remarkably, Winther and Gerdes [24] showed that two VapC from the entero-pathogenic bacteria *Shigella flexneri* and *Salmonella enterica* are specific tRNases that cleave the initiator tRNA. Thus, VapC toxins appear to target RNA by distinct mechanisms. Sequence specificities of both VapC1 and VapC19 from *M. tuberculosis* were recently investigated by mass spectrometry of mRNA fragments [26]. Both toxins appeared to be redundant, cleaving preferentially GC rich 4mers, with other sequences being cleaved less efficiently. Considering the fact that *M. tuberculosis* has a GC rich genome, with a GC content of 66%, these findings suggest that VapC cleavage sites are frequent in mRNA, and, thus activation of these toxins is likely to rapidly trigger a general translation inhibition. A recent analysis of VapBC20 revealed that the VapC20 toxin specifically inhibits translation by cleavage of the conserved Sarcin-Ricin loop of the 23S rRNA. In this case, the structure of the loop was essential for recognition and cleavage by VapC20 [27].

Structural analysis of the VapBC5 complex from *M. tuberculosis* revealed an asymmetric complex with a 1:1 stoichiometry [23]. However, the recently obtained crystal structure of the VapBC3 complex showed a different picture, with a toxin-antitoxin hetero-octamer formed by assembly of two heterotetramers containing two toxins and two antitoxins, similar to VapBC structures from other species, namely *Nisseria gonorrhoeae* FitAB, *Shigella flexneri* VapBC, and *Rickettsia felis* VapBC2 [28]. In spite of such differences, the two mycobacterial VapC toxins present a similar putative catalytic site formed by the conserved acidic residues of their PIN domains that coordinate the Mg^2+^ ion. In addition, both structures suggest a conserved mechanism of inhibition, in which the VapB antitoxins prevent efficient binding of Mg^2+^ at the active sites of their cognate VapC [23,28]. The *M. tuberculosis* VapB antitoxins are generally related to families of transcriptional regulators or DNA binding domains already reported to be associated with VapC toxins, *i.e.*, 33 RHH, 8 Phd, 2 ArbR, and 1 MerR [29]. However, five other VapB do not present any conserved domain.

Several VapBC of *M. tuberculosis* were found to be induced in response to relevant stress conditions encountered during the infection process [16]. This includes hypoxia (VapBC15, VapBC7, and VapBC25) and in IFN-γ-stimulated murine bone marrow-derived macrophages (VapBC11, VapBC3, and VapBC47). In addition, VapBC3, VapBC31, and VapBC49 were shown to be among the TA systems the most up-regulated in drug-tolerant bacteria, suggesting a possible role in persistence [12]. Interestingly, a recent study in the closely related bacterium *M. smegmatis* showed that its sole VapC toxin is an RNase that couples the rate of glycerol utilization to bacterial growth via post-transcriptional regulation of genes of sugar transport pathways [30]. In *M. tuberculosis*, it is conceivable that the multiple VapC could act as regulators of translation in response to a plethora of environmental stresses. Intriguingly, the extra cellular concentration of eleven VapC toxins from *M. tuberculosis*, namely VapC4, VapC5, VapC13, VapC19, VapC22, VapC27, VapC37, VapC39, VapC38, VapC41, and VapC44, was increased in a nutrient starvation model, further suggesting a possible role for these toxins during the establishment of latent infection [31].

## 3. The MazEF Family

Overexpression of the *E. coli* sequence-specific endoribonuclease MazF induces a growth arrest *via* cleavage of almost all cellular mRNAs in a ribosome-independent manner [32]. The *E. coli* MazEF complex in which the toxin MazF is inactive forms a linear heterohexamer composed of a dimer of the MazE antitoxin symmetrically bound to two MazF dimers [33]. Similar organization and stoichiometry is observed for the MazEF complex of *Bacillus subtilis*, although the MazE antitoxins are poorly related and present different fold [34]. Several MazF cleavage sites in mRNA have been reported, for example MazF from *E. coli* cleaves at ACA sequences [35] and the one from *B. subtilis* at UACAU [36]. Out of the 10 MazF toxin members present in *M. tuberculosis*, seven were shown to affect *E. coli* and/or *M. smegmatis* growth, two exhibited non-noticeable phenotype, and one has not been tested (Figure 1) [15,16,37]. Analysis of cleavage sites in mRNA, which has been performed for several MazF toxins of *M. tuberculosis* revealed significant differences. Indeed, while MazF9 cleaves at UAC sequences, MazF3 cleaves U rich regions [37], MazF6 at UUCCU sequences [37,38], and MazF4 at UCGCU [39]. Such differences in substrate recognition suggest that MazF toxins may trigger a large panel of responses, going from a global translation inhibition to a fine-tuned regulation of specific mRNAs. For example, in the case of MazF6, 31% of the total mRNA do not present the recognition site and are, thus, predicted to be resistant to toxin cleavage.

In addition to mRNA recognition, certain MazF toxins were shown to target ribosomal RNA. It is indeed the case for the *E. coli* MazF toxin, which generates specialized ribosomes by cleavage of the 3’ region of the 16S rRNA containing the anti-Shine-Dalgarno sequence, thus, facilitating leaderless mRNAs translation [40]. This mechanism would lead to an alternative translation pathway, which might promote stress adaptation. The MazF6 toxin from *M. tuberculosis* also acts on ribosomal RNA but by a very distinct mechanism. In this case, the toxin cleaves the 23S rRNA of dissociated ribosomes and subsequently provokes a global inhibition of protein synthesis [38]. Aside from RNA targets, it has been shown that the mycobacterial MazF4 toxin interacts with DNA topoisomerase I and that they mutually inhibit each other [17]. As the DNA topoisomerase I gene is essential in *M. tuberculosis* [41], this suggests that MazF4 could act by two alternative mechanisms to inhibit bacterial growth.

Expression of *mazEF* from *E. coli* is induced under various stress conditions, including DNA damage, heat shock and oxidative stress in a RelA dependent manner [42]. Similarly, it has been shown that *M. tuberculosis* MazF2 was down-regulated after four hours of nutrient starvation and MazF3 up-regulated during amino acid starvation in a *relA* deleted strain [43,44], thus, suggesting that MazEF modules from *M. tuberculosis* could also be regulated by the stringent response. As observed for RelE toxins (see next part), overexpression of either MazF1 (Rv2801c), MazF5 (Rv1942c), or MazF6 (Rv1102c) from *M. tuberculosis* induced drug-specific effects on the formation of persister cells (data not shown from [18]). Correspondingly, individual deletion of each of these three MazF encoding genes from *M. tuberculosis* differentially diminished the formation of drug-tolerant persisters [18]. Although not yet studied in detail, these data point towards an implication of MazEF modules in the formation of *M. tuberculosis* persisters in response to antibiotic treatment.

Partner specificity among TA pairs in *M. tuberculosis* has been studied as well. The presence of 79 possible TAs in this bacterium represents a unique system to address possible cross-talks, overlaps, or cooperation between TA systems. Remarkably, the *M. tuberculosis* MazF9 toxin was shown to be neutralized by the non-cognate antitoxins MazE6, VapB27, and VapB40 [19]. Such overlap is in agreement with the very low conservation observed between the different MazE antitoxins in this bacterium (4% to 22% of identity) and with the fact that VapB27 and VapB40 are closely related to the *E. coli* MazE. Accordingly, the non-cognate antitoxins MazE1 to 7 and MazE9 could neither interact with MazF6 nor alleviate its toxicity [45]. In contrast, *M. tuberculosis* MazF toxins are well conserved with up to 46% sequence identity. Together, these findings suggest that intricate networks of TA systems may exist in this pathogen.

## 4. The RelBE and YefM/YoeB Families

Mutations in the *relB* gene in *E. coli* were originally identified as conferring a delayed relaxed phenotype of the stringent response to amino acid starvation [46]. Later, it was shown that this phenotype was due to destabilization of the RelB antitoxin, leading to hyperactivation of the toxin RelE [47]. The YefM/YoeB family, named after its *E. coli* member, was first described as homologous of the Axe-Txe addiction system of the Enterococcus faecium plasmid pRUM [48]. The YefM antitoxins are homologous to Phd, the antitoxin of the Phd-Doc module of phage P1, whereas the YoeB toxins belong to the RelE superfamily [1]. Both RelE and YoeB toxins from *E. coli* are ribosome-dependent ribonucleases that cleave mRNA at the ribosomal A site [32]. Nevertheless, despite sequence and structural homology, *E. coli* RelE and YoeB seem to act by distinct mechanisms: RelE binds to the 30S subunit of 70S ribosomes and inhibits translation elongation, whereas YoeB binds to the 50S subunit and inhibits translation initiation [32]. RelE from *E. coli* was also shown to cleave tmRNA, thus, suggesting delicate interplay between such toxins and quality control mechanisms in response to amino acid starvation [49].

*M. tuberculosis* encodes for two RelBE systems, namely RelBE1 (Rv1247c-Rv1246c) and RelBE2 (Rv2865-Rv2866), and one YefM/YoeB (Rv3357-Rv3358). Note that the YefM/YoeB module discussed here was previously annotated as RelEB3, due to the fact that the toxin belongs to the RelE superfamily of ribonucleases. Yet, the YefM/YoeB (RelBE3) proteins from *M. tuberculosis* are very close to those of the *E. coli* YefM/YoeB system (58% and 68% sequence similarity respectively), with the mycobacterial toxin containing the conserved C-terminal histidine and tyrosine residues of YoeB involved in RNAse activity and not present in RelE [50,51]. Therefore, in contrast with RelBE1 and 2, the RelBE3 system clearly belongs to the YefM/YoeB family.

Previous gel filtration analyses of the *M. tuberculosis* YefM/YoeB complex suggested that it may form a heterotrimeric complex, as it is the case for the *E. coli* homologs [52]. However, structural analysis of both RelBE2 and YefM/YoeB revealed very similar heterotetrameric complexes composed of two heterodimers of one toxin bound to one antitoxin, with tetramerization occurring mainly via interaction between the two antitoxins [53]. Interestingly, while both RelE2 and YoeB toxins showed characteristic folds of RelE-like toxins [53], the structure of their respective antitoxins RelB2 and YefM were related the YefM/Phd antitoxins and not to either the *E. coli* or the archeal RelB [53]. As for YoeB, inactivation of *E. coli* RelE upon binding to RelB seems to occur via a conformational shift in the catalytic site of the toxin [50,54].

The two RelBE and the YefM/YoeB systems of *M. tuberculosis* were shown to be functional TA systems in *E. coli*, *M. smegmatis*, and in *M. tuberculosis* [18,55]. In agreement with their different mechanisms of action, the toxin YoeB was less toxic than RelE1 and RelE2 when expressed in *M. smegmatis* [53]. Similar results were obtained in *E. coli*, where YoeB expression only weakly affected colony-forming [47]. Intriguingly, Yang and colleagues [56] proposed that there might be complex interplays between these three systems with different patterns of cross interaction and promoter regulation. For example, they showed that YefM could interact only with its cognate toxin YoeB, whereas RelB1 and RelB2 were capable of interacting with any of the RelE1, RelE2, or YoeB toxins. Moreover, YefM is able to bind its promoter on its own, whereas RelB1 and RelB2 need to be part of the TA complex, but not necessarily with their cognate toxin [56]. Such multiple ways to inactivate toxins or to regulate expression of TA genes further emphasizes the complexity of toxin-antitoxin networks in this bacterium.

Transcriptional analyses revealed that RelBE2 is among the 10 most induced TA systems in *M. tuberculosis* drug-tolerant persisters [12]. Furthermore, while the *yefM/yoeB*, *relBE1*, and *relBE2* genes were all expressed during bacterial growth in Luria-Bertani medium, only *relE1*, *relB2*, and *yoeB* transcripts could be detected in human macrophages at late stages of infection [55]. All three *relE1*, *relE2*, and *yoeB* toxin-encoding genes were also up-regulated in response to antibiotic treatment, as well as in lung tissues of infected mice [18], thus, suggesting that such toxins could contribute to persistence. Accordingly, overexpression of each of the three toxins also led to an increased level of drug-tolerant persisters and the deletion of either *relE2* or *yoeB*, but not *relE1*, reduced the formation of persisters by four- to nine-fold [18]. However, none of the toxin mutants was affected for survival in mice model, indicating that the toxins may not individually contribute to infection *in vivo* [18].

## 5. The HigBA Family

The first identified *higBA* locus (host inhibition of growth) originates from the Rts1 plasmid of *Proteus vulgaris* where it functions as an addiction module [57]. TA members of this family encode- for a RelE-like toxin directly followed by an antitoxin that contains a HTH Xre-domain [29,58]. The HigB toxin from Rts1 plasmid was shown to act as a ribosome-dependent ribonuclease with a mechanism distinct from RelE and YoeB, in which the toxin binds the ribosome 50S subunit and cleaves preferentially at AAA sequences on the processing mRNAs [59]. HigB1 and HigB2 from *V. cholerae* were also studied and showed different, less specific cleavage patterns [60]. The recently solved crystal structure of HigBA from *P. vulgaris* revealed a heterotetrameric HigB-(HigA)2-HigB complex in which two HigA-HigB heterodimers are assembled via additional HigA-HigA interactions [61]. The structure of HigA in such complex was very similar to that of previously identified HigA-like antitoxin structures obtained in the absence of toxin (*i.e.*,: HigA from *Coxiella burnetii*, PDB entry 3trb and YddM from *E. coli* CFT073, PDB entries 2ICT and 2ICP; [62]), thus, indicating that HigA may not undergo major conformational changes upon toxin binding. Remarkably, the structure also shows that inactivation of HigB may not occur by direct occlusion of its active site by HigA, thus suggesting a novel mechanism of inhibition in which the large HigB-(HigA)2-HigB complex might sterically inhibit interaction of the toxin with ribosome-bound mRNA, as proposed by the authors [61].

*M. tuberculosis* H37Rv possesses three TA systems homologous to HigBA, *i.e.*, two classical two-component TA pairs HigBA2 (Rv2022c-Rv2021c) and HigBA3 (Rv3182-Rv3183) and one atypical tripartite system, named TAC (Toxin-Antitoxin-Chaperone), composed of a HigBA1 pair (Rv1955-Rv1956) coupled to a molecular chaperone Rv1957 (Figure 1). To date, nothing is known about the involvement of HigBAs in *M. tuberculosis* pathogenesis. However, it has been shown that both *higBA1* and *higBA2* locus, which are located on the same genomic island comprising other genes potentially involved in dormancy, are among the 10 most up-regulated TA systems in *M. tuberculosis* drug-tolerant persisters [12,63]. While the effect of HigB2 toxin on bacterial growth was not investigated yet, conditional expression of HigB3 did not significantly affect *M. smegmatis* growth (Figure 1) [16]. In contrast, HigB1 exhibits a robust toxicity both in *E. coli* and in mycobacteria, and has been more extensively studied as part of the TAC system.

## 6. The Tripartite TAC System

**Figure 2:**
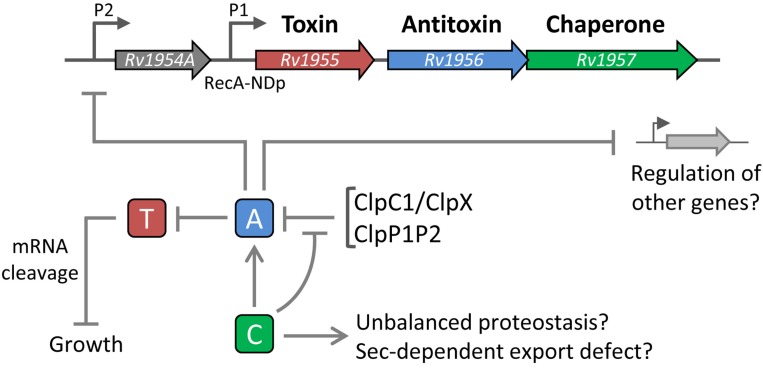
Proposed mechanism for TAC. In *M. tuberculosis*, the *TAC* genes *Rv1955-Rv1956-Rv1957*, respectively, encode the toxin HigB1 (T), the antitoxin HigA1 (A) and the chaperone Rv1957 (C). They are located within an operon also containing the less conserved upstream *Rv1954A* gene of unknown function. Expression of TAC is regulated by two promoters: the promoter P1, which contains a RecA-NDp motif characteristic of LexA/RecA-independent genes in *M. tuberculosis* and the promoter P2, recognized by the HigA1 antitoxin. Repression of the *TAC* operon by HigA1 could occur in complex with its HigB1 and/or Rv1957 partners and HigA1 could regulate other genes as well. The SecB-like chaperone Rv1957 facilitates the folding of HigA1 and prevents its degradation by proteases (potentially ClpC1 or ClpX together with the ClpP1ClpP2 proteolytic subunit). Although interaction with the chaperone renders HigA1 competent to neutralize the toxin, it is not known whether Rv1957 is part of the final inactive complex. Activation of the HigB1 toxin in response to stress, which induces growth inhibition through mRNA cleavage, mostly likely requires chaperone unavailability and subsequent degradation of the antitoxin, perhaps triggered by recruitment of the chaperone for Sec-dependent functions or by unbalanced proteostasis.

The TAC (toxin-antitoxin-chaperone) system from *M. tuberculosis* is encoded by three genes organized in operon, *Rv1955-Rv1956-Rv1957*, respectively encoding the HigBA1 TA pair (*Rv1955-Rv1956)* and a chaperone *Rv1957* related to the generic export chaperone SecB [64]. The operon also contains the upstream less conserved *Rv1954a* gene of unknown function. It has been shown that HigBA1 of TAC is specifically controlled by Rv1957 through a direct interaction between the chaperone and the antitoxin, allowing antitoxin folding and protection from degradation (Figure 2) [64]. Accordingly, expression of the HigA1 antitoxin does not counteract the severe toxicity induced by HigB1 in the absence of the chaperone, both in *E. coli* and mycobacteria [64]. In addition, single deletion of *higA1* in *M. tuberculosis* is lethal [65], while disruption of *Rv1957* alone exhibits a slow growth phenotype most likely due to a reduced antitoxin activity [41]. Therefore, it is very likely that the HigB1 activation cascade is triggered either by a decrease in expression or by direct hijacking of the chaperone at the post-translational level (Figure 2).

TAC is conserved in all members of the *M. tuberculosis* complex and a recent evolutionarily study showed that SecB-like chaperones associated with toxin-antitoxin systems can be found in many bacterial phyla [66]. Remarkably, this analysis also revealed that the presence of a chaperone was not restricted to HigBA TA pairs [66]. In addition, some bacteria, including *M. smegmatis*, only possess an antitoxin/chaperone pair, possibly due to the loss of the upstream toxin gene [66].

As stated above, expression of the HigB1 toxin in the absence of HigA1 and Rv1957 severely inhibits *E. coli*, *M. smegmatis, M. marinum*, and *M. tuberculosis* growth (Figure 2) [15,16,67]. A recent study performed in *M. tuberculosis* Δ*TAC* mutant revealed a general decrease of over 30 transcripts upon HigB1 overexpression, whereas only a limited number of genes were found induced in this context [67]. Strikingly, most of the dysregulated mRNA transcripts were putative targets of the iron- and zinc-transcriptional regulators IdeR/Rv2711 and/or Zur/Rv2359, known to limit the expression of iron and zinc uptake systems in conditions of high iron and zinc amounts, respectively. Therefore, these results suggest that HigB1 could respond to iron and/or zinc overload, and allow the rapid degradation of metal uptake systems, once the bacterial cell is replenished in iron and/or zinc, in order to avoid intoxication [68].

Transcription of the *TAC* operon is induced by several relevant stress conditions including DNA damage [69], heat shock [70], nutrient starvation [43], hypoxia [16], drug-persistence [12], and in host phagocytes [71]. The *TAC* operon is under the control of two known promoters: the *higB1*P1 located 51 nucleotides upstream of the start codon of *higB1*, thus, controlling the expression of *higB1*-*higA1*-*Rv1957*, and the more distal promoter *higB1*P2 located 29 nucleotides upstream of the start codon of the upstream *Rv1954a* [69]. Noticeably, the DNA-damage inducible P1 promoter possesses a RecA-NDp motif characteristic of LexA/RecA-independent genes in *M. tuberculosis* [69]. As observed for most TA loci, *TAC* is autorepressed by HigA1 binding to a palindromic motif overlapping the -35 element of the *higB1*P2 promoter [65]. Although very likely, it is not known whether HigB1 and/or Rv1957 also participate in this process (Figure 2).

A role for HigA1 as a global transcriptional regulator has been proposed as well. Indeed, microarray analysis of the Δ*TAC* mutant revealed that the *fadB*, *Rv3173c* and *Rv3662c* genes might be regulated by HigA1 [65]. Moreover, a one-hybrid reporter system showed that HigA1 is capable of interacting specifically with the promoter region of gene clusters containing stress response genes *whiB7*, *rubA*, *rubB*, and fatty acid metabolism genes *echA20*, *fadE28*, *fadE29*, *ufaA2*, and *fad26* [72]. Finally, ChIP-Seq analysis using FLAG-tagged HigA1 expressed in *M. tuberculosis* H37Rv revealed at least 30 potential HigA1 binding sites in intergenic regions, including the *higBA3* locus described above (TB Database sever). These data suggest that HigA could be part of a network of stress-associated regulons involved in *M. tuberculosis* pathogenesis.

As stated above, Rv1957 shares sequence similarities with the export chaperone SecB known to facilitate protein export in most Gram-negative bacteria. In *E. coli*, the homotetrameric SecB chaperone binds unfolded presecretory proteins, maintains them in a protected yet nonnative state and passes them to the SecA subunit of the Sec translocase, thus facilitating their export [73,74,75]. Remarkably, it has been shown that Rv1957 can functionally replace SecB during protein export *in vivo* in *E. coli* and prevent aggregation of the outer membrane protein precursor proOmpC *in vitro* [64]. Similarly, the SecB-like protein SmegB from *M. smegmatis* was also capable of performing such SecB-like function in *E. coli* [66]. The presence of a well-defined outer membrane and a remarkably large number of putative outer membrane proteins in *M. tuberculosis* and *M. smegmatis* [76,77,78] suggests that these bacteria could make use of a functional export chaperone under certain circumstances (Figure 2). Although nothing is known yet about such a role in protein export, an attractive possibility is that SecB-like chaperones specifically connect the activation of stress-responsive TA systems to the export process. Under certain stress affecting the Sec translocon, the cytoplasmic accumulation of certain presecretory proteins could thus compete with HigA1 for binding to the Rv1957 chaperone, facilitating degradation of the free antitoxin and the subsequent activation of the toxin (Figure 2). The relatively well-conserved association of SecB-like chaperones with stress-responsive TA systems, including members of the HigBA, HicAB, and MqsRA families [66] further suggests that the control of TA activation by molecular chaperones might represent a more commonly used mechanism to control bacterial growth in response to environmental changes affecting protein homeostasis. In agreement with such a hypothesis, it was recently shown that the major *E. coli* stress chaperone DnaK (HSP70) interacts with at least four antitoxins *in vivo*, namely MazE, RelB, MqsA and DinJ, whose respective toxin partners MazF, RelE MqsR, and YafQ were shown to affect translation [79]. Therefore, DnaK could control the activation of several TA systems in a manner comparable to that of Rv1957 from TAC. Further reinforcing the potential link between molecular chaperones and toxin activation, an independent study showed that overexpression of the HipA toxin of the type II TA system HipAB facilitates *E. coli* adaptation and survival in response to a severe proteotoxic stress induced by the lack of both major chaperones Trigger Factor and DnaK [80]. More work is warranted to elucidate such fundamental links between networks of stress-induced molecular chaperones and TA systems in bacteria.

## 7. The ParDE Family

*M. tuberculosis* H37Rv possesses two ParDE systems (Figure 1). ParDE was originally discovered on the broad-host-range plasmid RK2 as a stabilization system [81]. The ParE toxin does not target RNA but acts by inhibiting the DNA gyrase, thereby blocking DNA replication [82]. The crystal structure of the ParDE1 complex from *Caulobacter crescentus* revealed a heterotetrameric complex composed of two homodimers of toxin and antitoxin [54]. As *C. crescentus* ParD1, the ParD1 antitoxin from *M. tuberculosis* is predicted to have an RHH DNA binding domain, whereas ParD2 does not seem to contain any conserved domain. Ectopic expression of the ParE2 toxin from *M. tuberculosis* inhibited both *E. coli* and *M. smegmatis* growth, whereas, under the same conditions, ParE1 only affected *E. coli* growth [15,16]. Thus far, none of these systems were tested in *M. tuberculosis*.

## 8. Other TA Modules

*M. tuberculosis* H37Rv genome potentially encodes 11 TA systems that do not yet belong to canonic well-characterized TA families (Figure 1). Among these systems, three were experimentally tested and two were functional in *M. smegmatis* (Figure 1) [16]. Four of these TA modules were identified as part of the 10 most induced TA systems in drug-tolerant persister cells, however, none of them have been tested for TA functions (Figure 1) [12]. Except for the Rv2653c-Rv2654c system, all these TA pairs contain a conserved domain in the toxin and/or in the antitoxin. These modules are paired together as follows, with the toxin domain designated first (NCD stands for No Conserved Domain): PF10604-PF14013 (Rv0910-Rv0909), PF10604-NCD (Rv1546-Rv1545), GNAT-RHH (Rv0919-Rv0918), COG5654-Xre (Rv1989c-Rv1990, Rv3189-Rv3188), COG3832-ArsR (Rv2035-Rv2034), NCD-COG2110 (Rv0059-Rv0060), DUF1814-COG5340 (Rv1045-Rv1044, Rv2826c-Rv2827c), DUF1814-COG4861 (Rv0836c-Rv0837c). Note that all of these TA domain pairs have been predicted either by Makarova *et al*. [29], Sberro *et al*. [83], or Dy *et al*. [84].

In the case of the DUF1814-COG5340 (Rv1045-Rv1044, Rv2826c-Rv2827c) TA pairs, homologous systems from diverse bacteria were experimentally tested and indeed behaved as *bona fide* TAs [83]. This includes the MosAT TA system involved in the maintenance of the SXT conjugative element in *Vibrio cholerae* [85] and the recently discovered lactococcal AbiE bacteriophage abortive infection system [84]. DUF1814 toxins belong to the polymerase β nucleotidyltransferase (polβ NTase) superfamily and, accordingly, it has been shown that the toxin of AbiE specifically binds GTP *in vitro* [84]. Remarkably, no interaction between the toxin and the antitoxin of AbiE could be detected by co-immunoprecipitation experiments performed in *E. coli*, thus, suggesting that conserved DUF1814-COG5340 pairs might belong to type IV TA systems [84]. This study also revealed that the DUF1814-COG4861 (Rv0836c-Rv0837c) pair might belong to the same AbiE family and thus potentially represents a third type IV member in *M. tuberculosis*.

## 9. Role of *M. tuberculosis* Proteases in TA Activation

Toxins from type II TA systems are generally activated following degradation of their cognate antitoxins. The major stress proteases Lon and/or ClpP together with its AAA^+^ subunit ClpX, ClpA or ClpC, were identified as key players in this process [86,87,88,89,90,91]. Although it clearly appears that certain antitoxins possess intrinsically flexible domains sensitive to proteolysis that are protected when bound to the toxin, very little is known about specific degradation signals within antitoxins that determine selective proteolysis [92,93,94]. Likewise, the cellular cascades that trigger activation of proteases towards TA systems are not well understood. In several cases, it has been shown that TA systems are induced in response to nutritional stress [86,87]. Such conditions are known to induce the stringent response characterized by the synthesis of the small alarmone molecule (p)ppGpp, which causes growth arrest, activation of the stress response, and bacterial persistence [95]. Remarkably, increased persistence in *E. coli* strongly relies on both the activation of Lon by inorganic polyphosphates known to accumulate in response to (p)ppGpp synthesis, and the presence of TA systems, as shown recently [8]. Although it has been shown that the stringent response is important for *M. tuberculosis* persistence, it is not known whether activation of TA systems by specific stress proteases contributes to such phenomenon [44]. 

In contrast with *E. coli*, *M. tuberculosis* and closely related species do not have the proteases Lon. However, they do have two *clpP* genes, *clpP1* and *clpP2*, encoding the proteolytic subunits and three predicted ClpP-associated regulatory subunits encoding genes, *clpX*, *clpC1*, and *clpC2*, involved in substrate recognition, unfolding and translocation into the proteolytic chamber [96]. Note that in contrast with ClpC1 and ClpX, the ClpC2 sequence does not contain any AAA^+^ domain and it is thus not clear whether it can cooperate with ClpP proteases. Yet, the most striking difference with *E. coli* is that *M. tuberculosis* possesses a 20S proteasome-like degradation machinery to which proteins are addressed by addition of an ubiquitine-like tag called Pup (Prokaryotic ubiquitin-like protein), giving the name of “Pupylation” to this pathway [97]. Analysis of the Pup proteome of *M. tuberculosis* revealed that at least 4 toxins, namely Rv0059 (unclassified), Rv0749 (VapC31), Rv2035 (Unclassified) and Rv2527 (VapC17) are pupylated, while no pupylated antitoxin could be detected [98]. Although there is no evidence for a role of the proteasome in TA activation, the observation that pupylation seems to concern only toxins suggest that an additional, yet unexplored level of regulation of TA systems by proteolysis could exist.

## 10. Concluding Remarks

In contrast with closely related mycobacteria, *M. tuberculosis* encodes a remarkably high number of TA systems, with at least 37 being functional *in vivo*. The existence of such intricate networks of TAs, often induced during persistence or under stress conditions encountered during the infection process, raises the question of their implication in the virulence of this dangerous pathogen. While ectopic expression of certain toxins inhibits *M. tuberculosis* growth and results in increased persistence, mutation of distinct TA genes did not yet reveal a direct involvement of these systems in the infection process *in vivo*. The fact that decreased persistence in *E. coli* could only be observed upon successive deletions of several TA systems strongly suggests that TA functions are redundant and that similar situation might occur in *M. tuberculosis*. Finally, even though the signals that trigger activation of toxins in *M. tuberculosis* are unknown, the observation that a single protease can induce persistence in *E. coli* via stochastic activation of several TA systems indicates that a search for specific stress proteases that target antitoxins in *M. tuberculosis* might reveal important links between TA activation and persistence in this bacterium. 
